# Supplementary material for: Biotinylation-dependent near-neighbor analysis for identification of septation regulators in Aspergillus fumigatus
Source: bioRxiv. 2026 Sep 18:2026.09.17.752449. Preprint. [Version 1] doi: 10.64898/2026.09.17.752449 (PMC13596442; doi:10.64898/2026.09.17.752449)

bioRxiv preprint doi: https://doi.org/10.64898/2026.09.17.752449; this version posted September 18, 2026. The copyright holder for this preprint (which was not certified by peer review) is the author/funder, who has granted bioRxiv a license to display the preprint in perpetuity. It is made available under a CC-BY 4.0 International license.

Supplemental Figure 1: Impact of *mobB* repression on colony morphology

10 µl of 10^6 conidia/ml of the indicated strain was inoculated onto GMM in a 6-well plate containing increasing concentrations of doxycycline. Plates were incubated for 2 d at 37°C.

Supplemental Figure 2: TurboID is highly expressed in pOtef-TurboID

RT-qPCR was performed using RNA extracted from overnight culture of the indicated strains. Expression was normalized to *tubA.* TurboID transcript level was compared to *pOtef-TurboID* by two-tailed T test. *: $p<0.05$. Data points are the average of two technical replicates of three biological replicates. C)

Supplemental Figure 3: Impact of *sepG* repression on colony morphology and septation

A) 10 µl of 10^6 conidia/ml of the indicated strain was inoculated onto GMM in a 6-well plate containing increasing concentrations of doxycycline. Plates were incubated for 2 d at 37°C.

Supplemental Figure 4: Hyphal septation of selected mutants

10^4 conidia of each strain was inoculated in 1ml GMM in a glass-bottomed 24 well plate and incubated for 16 h at 37°C. Wells were washed and stained with CFW to visualize septa.

Supplemental Figure 5: Induction of *sepG* expression restores septation

Conidia were inoculated into wells of a glass-bottomed 24-well plate in GMM supplemented with increasing amounts of doxycycline and incubated for 16 h at 37°C. Coverslips were stained with CFW and septa were imaged on an inverted fluorescent microscope using a DAPI filter.

Supplemental Figure 1

CEA10

0 0.25 0.5

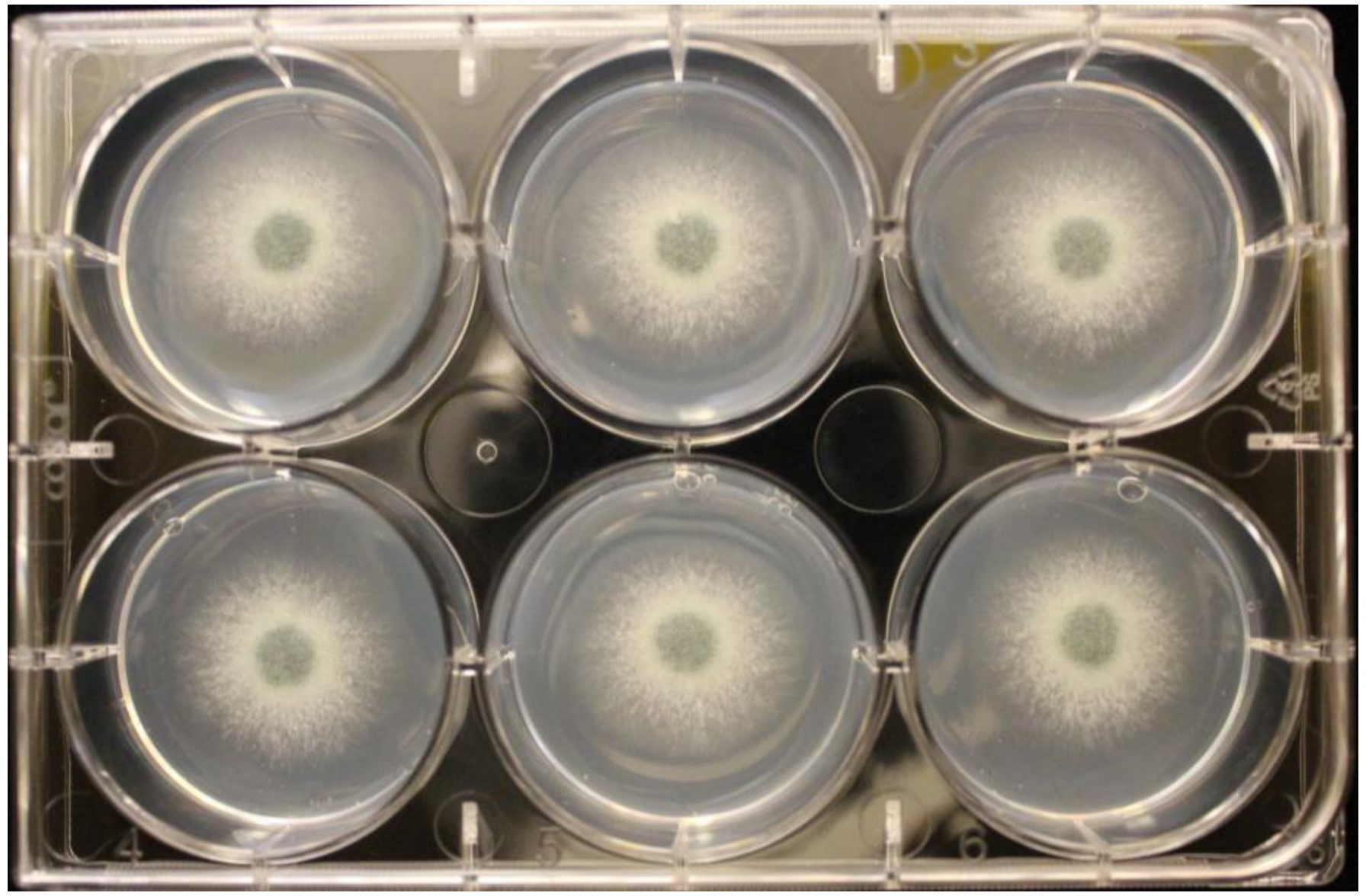

1.0 2.0 4.0

pTetOff-MobB

0 0.25 0.5

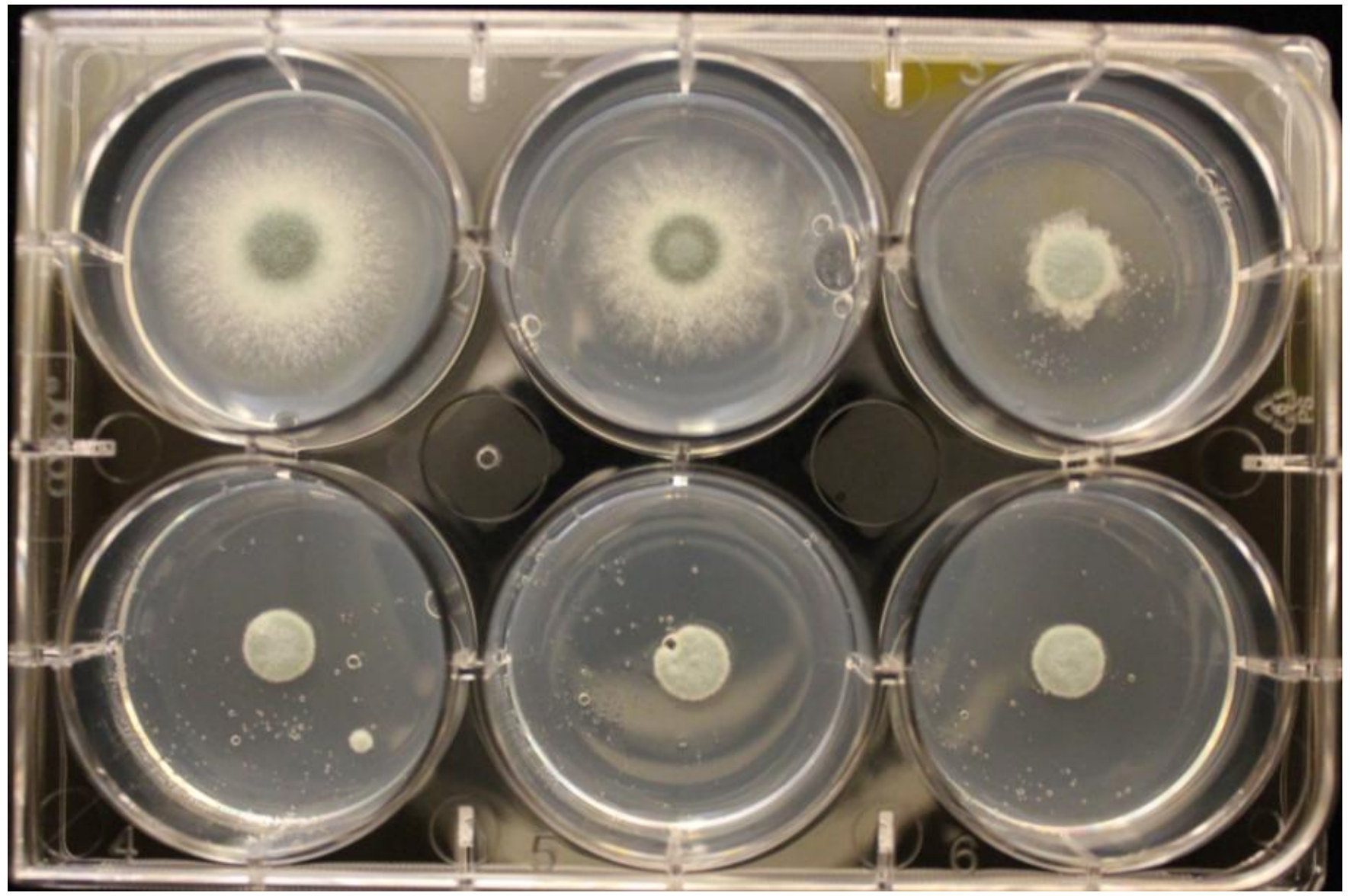

1.0 2.0 4.0

Supplemental Figure 2

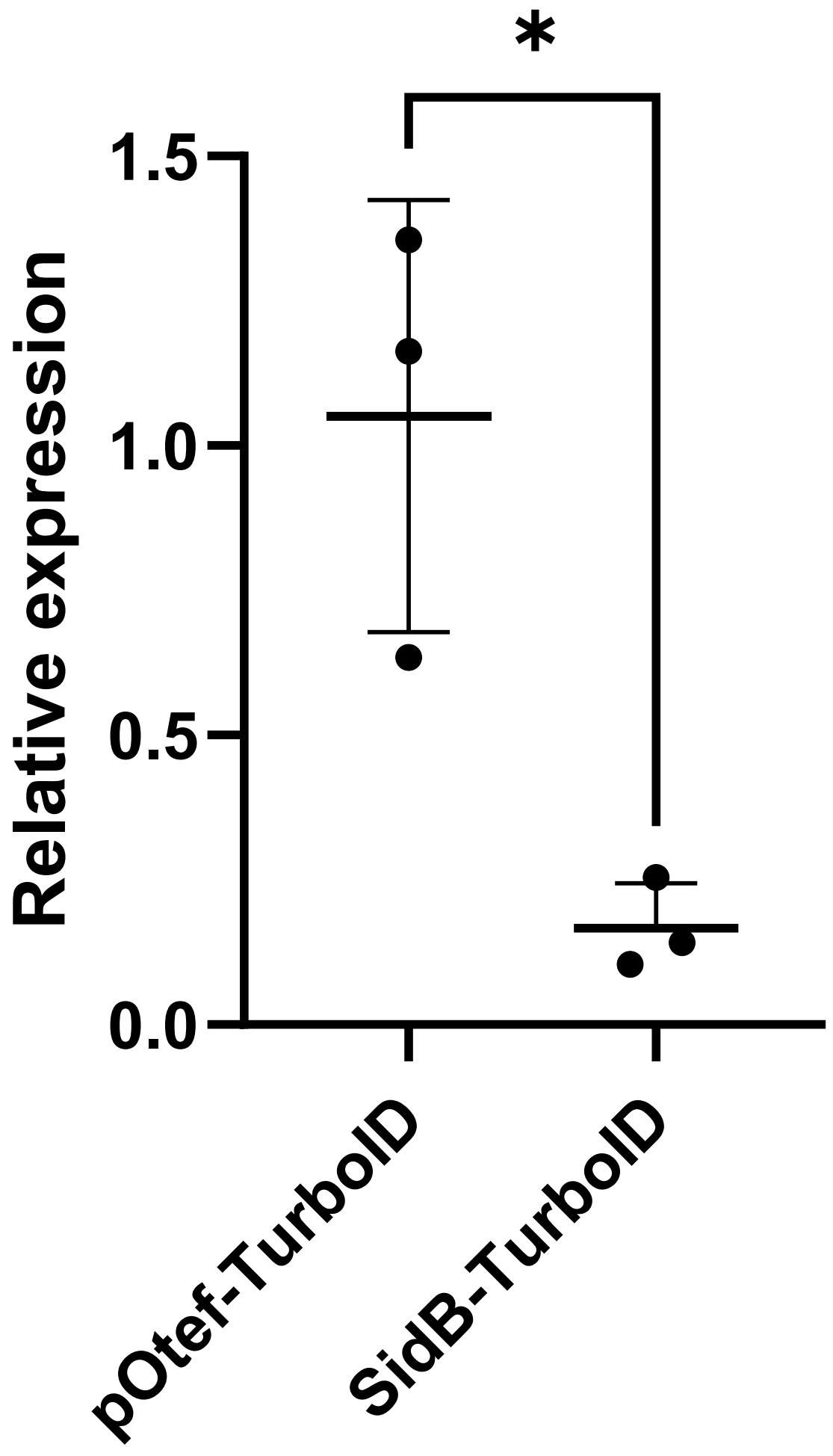

Supplemental Figure 3

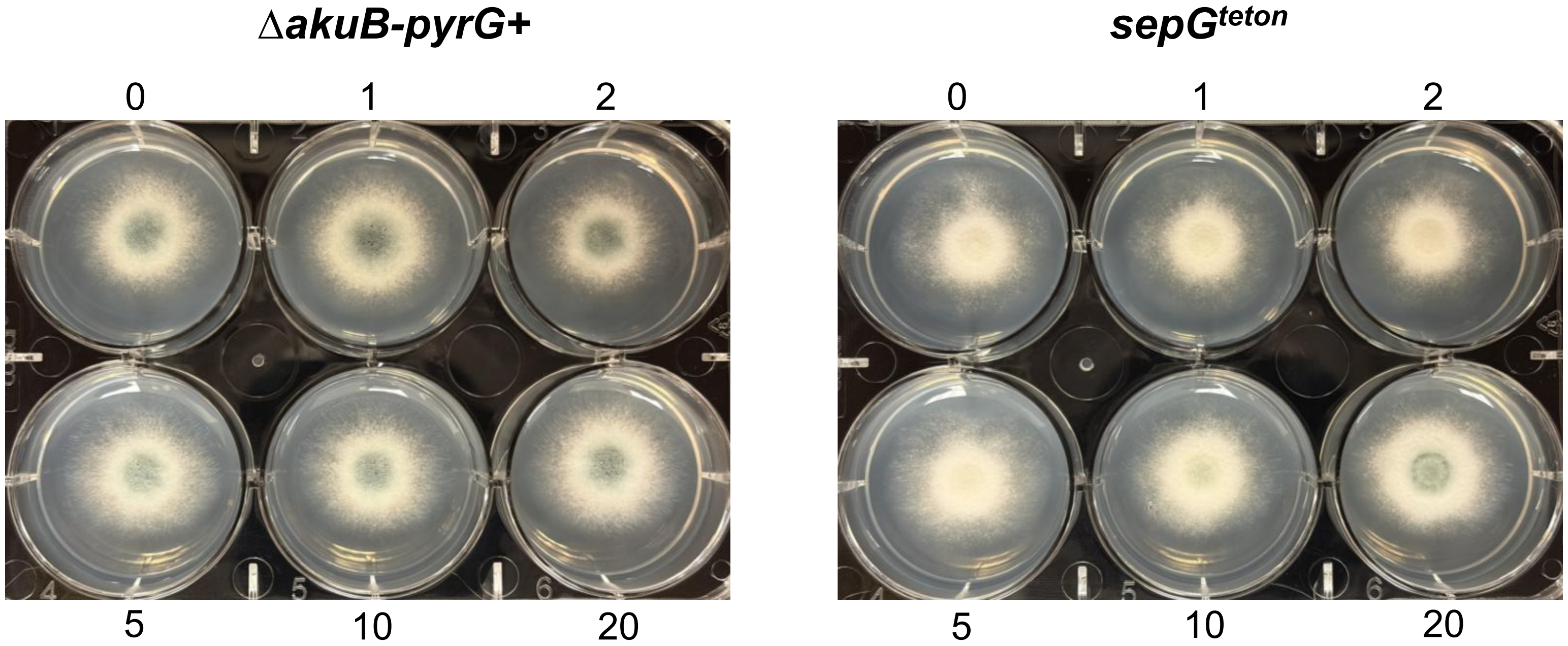

Supplemental Figure 4

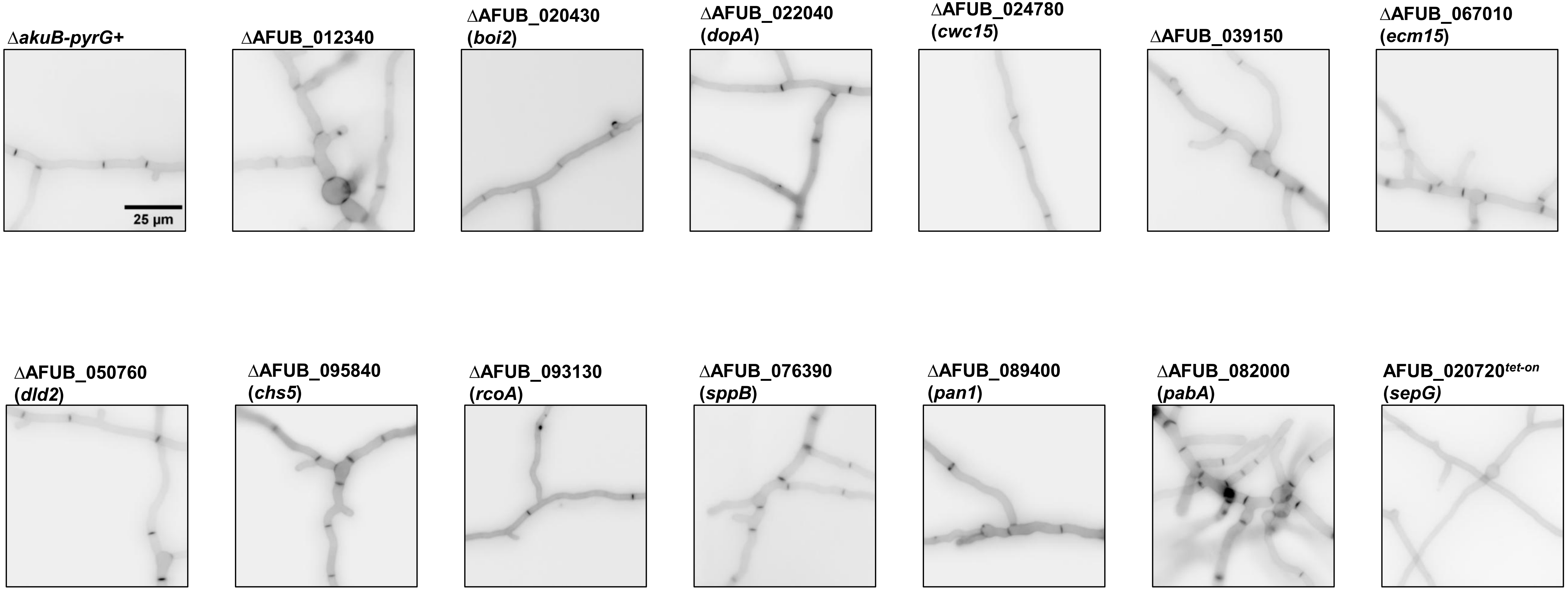

Supplemental Figure 5

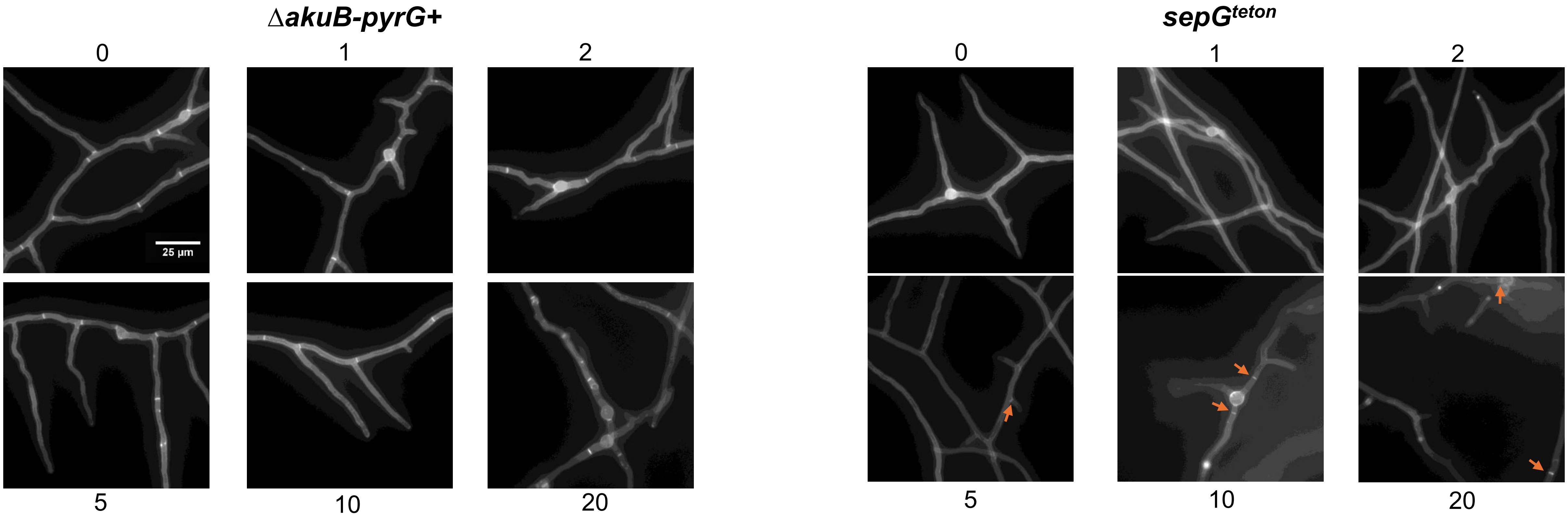

Supplement: Supplementary file 1 [file NIHPP2026.09.17.752449v1-supplement-2.pdf]
